# Effects of Nutrition Interventions on Athletic Performance in Soccer Players: A Systematic Review

**DOI:** 10.3390/life13061271

**Published:** 2023-05-28

**Authors:** Ines Aguinaga-Ontoso, Sara Guillen-Aguinaga, Laura Guillen-Aguinaga, Rosa Alas-Brun, Francisco Guillen-Grima

**Affiliations:** 1 Departament of Health Sciences, Public University of Navarra, 31008 Pamplona, Spain; sguillen.4@alumni.unav.es (S.G.-A.); rosamaria.alas@unavarra.es (R.A.-B.); 2 Area of Epidemiology and Public Health, Healthcare Research Institute of Navarre (IdiSNA), 31008 Pamplona, Spain; 3 Department of Nursing, Suldal Hospital, 4230 Sands, Norway; lguillen@alumni.unav.es; 4 Department of Preventive Medicine, Clínica Universidad de Navarra, 31008 Pamplona, Spain

**Keywords:** soccer performance, supplements, diet, systematic review, high carbohydrate diet, creatine supplementation, tart cherry, betaine, bicarbonate and minerals, professional players

## Abstract

Background: More than 270 million participants and 128,893 professional players play soccer. Although UEFA recommendations for nutrition in elite football exist, implementing these guidelines among professional and semiprofessional soccer players remains suboptimal, emphasizing the need for targeted and individualized nutritional strategies to improve adherence to established recommendations. Methods: We conducted a comprehensive search in PubMed, Scopus, Web of Science, and clinical trial registers. Inclusion criteria focused on professional or semiprofessional soccer players, nutrition or diet interventions, performance improvement outcomes, and randomized clinical trial study types. We assessed quality using the Risk of Bias 2 (RoB 2) tool. We identified 16 eligible articles involving 310 participants. No nutritional interventions during the recovery period effectively improved recovery. However, several performance-based interventions showed positive effects, such as tart cherry supplementation, raw pistachio nut kernels, bicarbonate and mineral ingestion, creatine supplementation, betaine consumption, symbiotic supplements, and a high-carbohydrate diet. These interventions influenced various aspects of soccer performance, including endurance, speed, agility, strength, power, explosiveness, and anaerobic capacity. Conclusions: Specific strategies, such as solutions with bicarbonate and minerals, high carbohydrate diets, and supplements like creatine, betaine, and tart cherry, can enhance the performance of professional soccer players. These targeted nutritional interventions may help optimize performance and provide the competitive edge required in professional soccer. We did not find any dietary interventions that could enhance recovery.

## 1. Introduction

Soccer, a sport enjoyed by over 270 million participants globally, including nearly 130,000 professional players, is an intermittent sport combining high-intensity and low-intensity activities [1,2,3,4]. Soccer players typically cover 10–14 km per match, with more than 8% being high-intensity running [5,6,7,8,9,10]. Although physical match demands for professional soccer players are well-studied, the physical demands of training, usually over short periods, have been less explored [11,12,13].

Optimal performance in soccer requires full physical fitness, strength, endurance, and agility, necessitating strategies to meet energy demands and enhance performance [14,15,16]. Failing to meet these demands can result in fatigue, cognitive dysfunction, weight loss, and increased susceptibility to injuries, among other adverse effects. Nutritional interventions are a potential strategy, but evidence of their impact on professional soccer players is limited.

Delayed Onset Muscle Soreness (DOMS), a type of muscle pain and stiffness that occurs 24–72 h post-intense or unfamiliar physical activity, is a challenge for athletes [17,18,19]. The condition is believed to be linked to microscopic damage to muscle fibers and connective tissue, resulting in an inflammatory response causing pain, swelling, and reduced muscle function [20,21]. DOMS is particularly prevalent during preseason and training, with intensive resistance and strength training exercises [22,23,24].

The relationship between nutrition and athletic performance has gained attention, with interventions like carbohydrate ingestion, dietary supplementation, and specific diet plans proposed to enhance athletic performance [25]. The International Olympic Committee (IOC) and other sports organizations have recognized ergogenic aids such as caffeine, creatine, nitrate, sodium bicarbonate, and beta-alanine as beneficial for athletes [26,27,28,29].

Despite published practical recommendations on nutrition for elite soccer players [30], many players fail to meet these nutritional guidelines, as indicated by a systematic review [29]. These findings highlight the importance of effective dietary strategies for performance optimization and recovery. Soccer players’ carbohydrate intake, crucial for peak performance during the competitive season, often falls below recommended values [30,31]. Therefore, personalized recommendations considering training periodization and field positions can support soccer players in adhering to guidelines and optimizing health and performance outcomes.

Sports like soccer and martial arts rely heavily on anaerobic capacity and repeated sprint ability, with fatigue often resulting from metabolite accumulation and energy substrate depletion [19,32,33,34,35]. Buffering agents such as sodium bicarbonate can counteract exercise-induced acidosis, enhance anaerobic performance, and improve lactate utilization [36,37]. The International Society of Sports Nutrition (ISSN) recommends sodium bicarbonate supplementation at 0.2 to 0.5 g/kg doses to enhance performance in high-intensity sports [38].

Recent attention has been drawn to raw pistachio nut kernels as a potential functional food for improving sports performance due to their high antioxidant content [39]. They are rich in phenolics, essential fatty acids, and micronutrients, potentially enhancing physical and cognitive functions for optimal soccer performance [40,41]. However, the exact effects of pistachios on exercise performance and redox status requires further research [42,43].

Betaine, found in sugar beets and spinach, is a byproduct of choline metabolism. It can stabilize native protein structures under stress and improve anaerobic performance by reducing cellular acidosis [44,45]. Studies have shown that betaine improves body composition and performance when incorporated into a resistance training program [46,47,48,49].

Tart cherry juice, rich in antioxidant and anti-inflammatory phytochemicals, can potentially aid recovery after strenuous exercise and maintain the inflammatory response and redox balance [50,51,52,53,54]. However, its effects on exercise-induced muscle damage (EIMD) and delayed-onset muscle soreness (DOMS) are mixed, with some studies reporting positive effects on muscle strength and inflammation and others finding no significant impact [55,56]. While it has shown positive effects on soccer players’ performance in lab conditions, its impact under normal game conditions on professional soccer players remains unclear [57].

Yohimbine, a stimulant and aphrodisiac traditionally used, affects the central and peripheral nervous systems by interacting with various monoaminergic receptors [58]. It has been investigated for potential benefits in conditions like erectile dysfunction, inflammatory disorders, and even in enhancing athletic performance [59,60]. However, it is associated with an incident where an athlete tested positive for a banned substance after ingesting yohimbine, raising questions about its safety and regulation in sports [61,62,63].

Despite the proposed benefits, the evidence does not support the isoprotein ketogenic diet as a performance enhancer. Studies found no performance improvement in athletes using this diet, and in some cases, it impaired anaerobic exercise performance [64,65].

Creatine is a popular ergogenic aid among athletes, known for enhancing performance and promoting exercise adaptations. It is particularly beneficial for short-duration, high-intensity exercises [66,67,68,69,70]. The International Society of Sports Nutrition (ISSN) suggests that healthy individuals can use creatine supplements safely, and recent evidence refutes the notion that they could lead to dehydration or muscle cramping [71,72]. Creatine users have shown a reduced risk of heat-related illnesses, and its supplementation can enhance high-intensity exercise performance [73]. However, it does not appear to affect aerobic activities [74]. Single-dose ingestion of a creatine-based supplement has also shown benefits [75].

Synbiotics, combinations of probiotics and prebiotics, can benefit health, such as by modifying gut health [76,77,78,79]. Some studies have explored their potential effects on immunosuppression in football players [80,81].

Limited information exists on how nutrition impacts professional and semi-professional soccer players’ performance [82]. This systematic review aims to comprehensively understand how nutrition interventions may benefit these athletes and guide future research and practical applications, thus improving nutrition strategies in professional soccer.

## 2. Materials and Methods

### 2.1. Outcome

The expected outcome of this study is to review and analyze the influence of nutrition interventions on the performance of professional soccer players. The primary outcome of this study is to review and analyze the effectiveness of various nutrition interventions in improving the performance of professional soccer players in areas such as endurance, speed, agility, strength, power, explosiveness, and anaerobic capacity. In this study, our primary focus centers on athletes’ performance during the crucial preseason and ongoing training periods. By ‘performance’, we refer to the comprehensive spectrum of physical and skill-related capacities exhibited during these specific times. The preseason period, a significant preparatory phase leading up to competitive seasons, is critical for athletes to condition their bodies, enhance their skills, and mentally prepare for upcoming competitions. Concurrently, the training sessions across the year represent an essential platform for athletes to continuously maintain and advance their skill levels, physical condition, and competitive readiness. The measures and outcomes of these specific periods provide a broad and insightful lens into an athlete’s readiness, development, and potential for in-competition success. The secondary outcome is to evaluate the effectiveness of these interventions in enhancing recovery during the post-match period.

### 2.2. Design

We carried out this systematic review using the PRISMA recommendations and the guidelines of the Cochrane Handbook of Systematic Reviews [83,84]. The protocol for this systematic review can be accessed and provides detailed information on the methodology and search strategies employed [85].

### 2.3. Search Strategy

A systematic search was conducted in PubMed, Scopus, and the Web of Science (WOS), covering all studies published before 25 February 2023.

**Figure 1:**
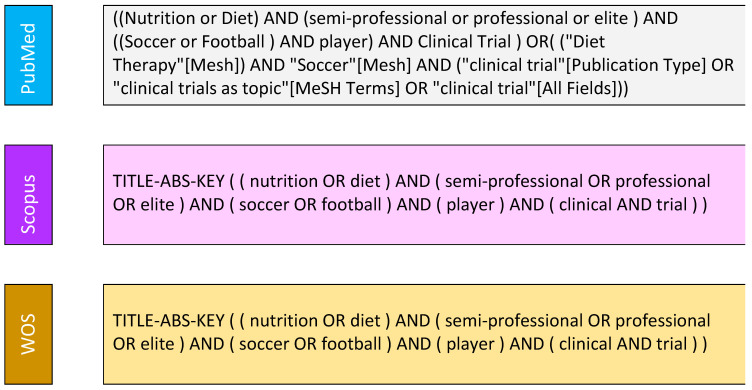
Descriptors employed in the systematic search on PubMed, Scopus, and WOS databases.

We searched clinical trial registers, including ClinicalTrials.gov, the WHO International Clinical Trials Registry Platform, and the European Clinical Trials Database. We also searched the web for clinical trials to identify relevant publications. We initially created search queries (Figure 1) to link generic keywords associated with clinical trial interventions on nutrition or diet for professional or semiprofessional soccer players. We applied no filters based on the players’ gender or age. When available, we included the publication of a clinical trial’s results in the review.

We searched Google Scholar for published articles of registered clinical trials not yet indexed in the databases (PubMed, Scopus, and WOS).

### 2.4. Inclusion and Exclusion Criteria

The inclusion criteria of this study were formulated according to the PICOS principles as follows: (1) P: The subjects were professional or semiprofessional soccer players. (2) I: The experimental group needs to use a nutrition or diet intervention. C: The control group required a different intervention (e.g., another supplement). (4) O: The outcomes were improvements in the players’ performance (without specific outcome measures specified). (5) S: The study type was randomized clinical trials.

We clearly distinguished between professional and semi-professional soccer players in defining our inclusion and exclusion criteria. This differentiation is pivotal to our study as it acknowledges the variance in the levels of physical preparation, training intensity, and body composition characteristics between these two groups. Professional players earn their living by playing soccer, signing club contracts, and being paid a regular salary. They are typically registered with a professional league or federation, as outlined by the FIFA regulations on the status and transfer of players. Professional soccer players train multiple times daily and maintain a body fat percentage typically not exceeding 10–11%, irrespective of the method used to predict fat [86,87].

In contrast, semi-professional soccer players who often play in lower-tier leagues and divisions may exhibit a more wide-ranging body fat percentage. This variability can be attributed to several factors, including less rigorous training regimens, dietary habits, and genetic predispositions [88]. On the other hand, semi-professional players do not depend solely on soccer for their income. While they may receive some form of compensation for playing, they usually have other sources of income and train less frequently, approximately two times a week.

We applied the following exclusion criteria: (1) repeated publication; (2) inability to obtain the full text; (3) incomplete or unavailable data; and (4) studies not published in Spanish or English. We excluded clinical trials that recruited participants or had not published results in clinical trial registries or scientific publications. We selected the most up-to-date information from the clinical trials registry and scientific publications, if available.

### 2.5. Data Extraction

We removed duplicate studies using EndNote (version 20; Clarivate Analytics, Philadelphia, PA, USA) [89]. The study selection process was counted with assistance from the Rayyan website and a mobile application [90]. Two reviewers (SGA and FGG) independently screened the titles, abstracts, and keywords. If a study was considered a candidate, the full text was independently assessed and evaluated based on inclusion and exclusion criteria. The third reviewer (LGA) resolved disagreements through consultation with the first two. The authors accessed the full text whenever a study met the inclusion criteria. After searching and evaluating complete articles, four authors (SGA, FGG, LGA, and IAO) independently reviewed the text of all selected studies to determine their inclusion. The authors resolved any differences in their selections through discussion. The writers consulted a fifth author (RAB) if they could not agree.

The information extracted from the articles contained: the author, year, country, participants, sample size in the experimental and control groups (E/C), intervention (E/C), length of intervention days, and outcomes.

### 2.6. Quality Assessment

We used the Risk of Bias 2 (RoB 2) tool, recommended by the Cochrane Systematic Review Manual (5.1.0) [84,91], to assess the risk of bias in the included literature. This tool was employed to strictly evaluate the quality of the literature. In crossover designs, we used the Risk of Bias tools—RoB 2 for crossover trials [92]. Two reviewers (IAO and LGA) conducted independent assessments of the risk of bias for each included article, categorizing them as “low risk,” “some concerns,” or “high risk.”. Another reviewer (RAB) reviewed the results and resolved any disagreements.

## 3. Results

### 3.1. Study Selection

**Figure 2:**
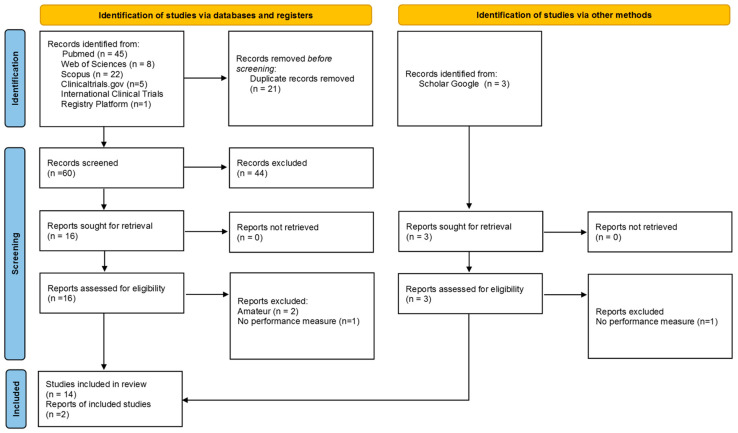
*PRISMA* 2020 flow diagram.

A database search identified 60 records, of which 14 articles met the eligibility criteria [93,94,95,96,97,98,99,100,101,102,103,104,105]. Web searching of clinical trials identified two additional articles [106,107]. Thus, 16 articles were included in this review [93,94,95,96,97,98,99,100,101,102,103,104,105,106,107]. The articles included in this study were published between 1992 and 2023. Figure 2 presents the study selection flow.

### 3.2. General Characteristics of Included Studies

**Figure 3:**
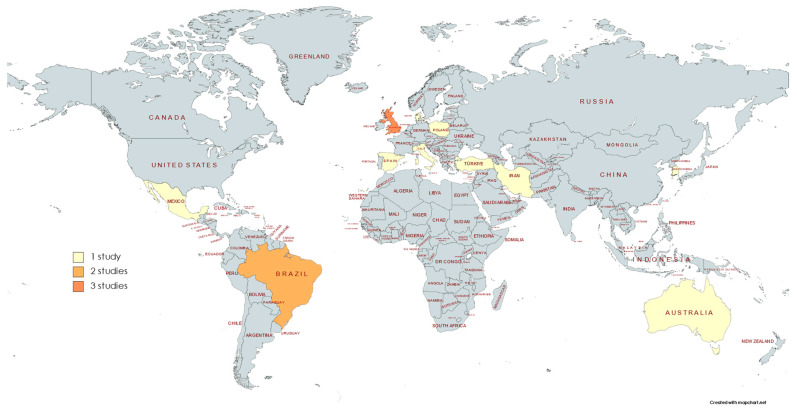
Geographic distribution of publications by country.

There were 11 parallel clinical trials [15,95,96,97,100,101,102,103,105,106,107] and 5 cross-over studies [93,94,98,99,104]. The included studies had publication dates ranging from 1992 to 2023, with a median publication year of 2017.5 and a quartile deviation (QD) of 7 years. Of the sixteen studies included, the participants were only female in one study [37], while the remaining fifteen were male players. There were studies on all the continents except Africa. In 68.8% of the studies, researchers conducted their investigations in Europe. The countries with more studies were the UK (3) and Brazil (2) (Figure 3).

Some studies used several groups, like sedentary people and soccer players. We only studied the soccer players group [103,107]. The total number of participants was 310. The studies were of reduced size, with a median of 18.5 participants (QD = 5). The participants in 3 studies were semiprofessional players, and in 12 studies, professional players.

### 3.3. Risk of Bias in the Studies

The Risk of Bias was evaluated separately for Parallel Clinical Trials and Cross Over studies.

#### 3.3.1. Parallel Clinical Trials

**Figure 4:**
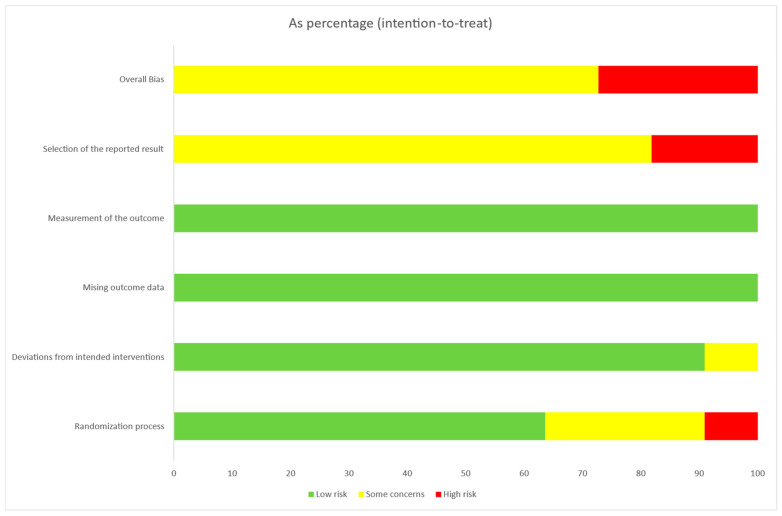
Risk of bias assessment in Parallel Clinical Trials (RoB2).

According to the RoB2 assessment of the risk of bias in RCTs, 30% of the studies showed a high risk of bias, 72.7% showed some concerns about the risk of bias (a moderate risk of bias), and no one showed a low risk of bias. Specifically, 90.9% of the studies had a moderate to low risk of bias in the randomization process, and 100% had a low risk of bias in the deviation from the intended interventions and missing outcome data. In the measurement of the outcome dimension, 81.8% had a low to moderate risk of bias in selecting outcomes, and 18.2% had a high risk (Figure 4).

**Figure 5:**
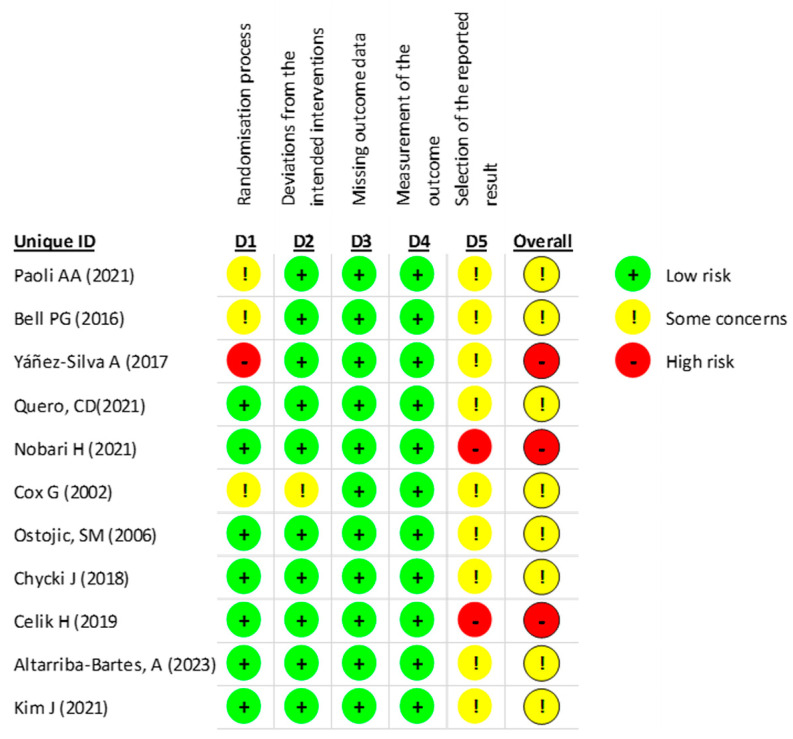
Risk of bias assessment for each parallel clinical trial included (RoB2) [15,95,96,97,100,101,102,103,105,106,107].

We present the evaluation of each study in Figure 5.

#### 3.3.2. Cross Over

**Figure 6:**
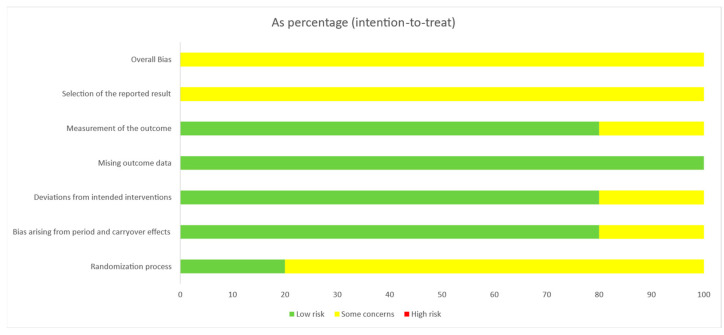
Risk of bias assessment in crossover studies (RoB2).

**Figure 7:**
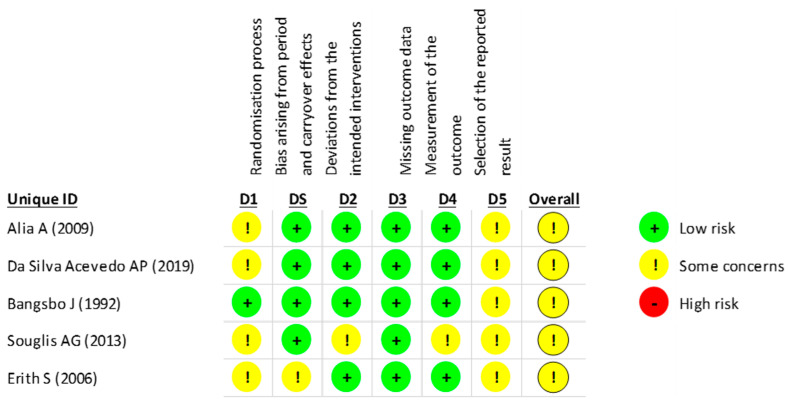
Risk of bias assessment for each crossover study included (RoB2) [93,94,98,99,104].

In the RoB2 assessment of the risk of bias in crossover studies, 100% of the studies showed some concerns about the risk of bias (moderate risk of bias). Specifically, 20% of studies had a low risk of bias in the randomization process, and 80% had a low risk of bias that arose from period and carryover effects and the deviation from the intended interventions and measurement of the outcome. There was a low risk of bias from missing outcome data in 100% of the studies. The “selection of the reported result,” dimension was 100% moderate (Figure 6). The evaluation of each crossover study is shown below (Figure 7).

### 3.4. Intervention Strategies

Our review includes a range of studies examining various nutritional interventions, such as carbohydrate ingestion, supplementation with creatine, betaine, yohimbine, and other compounds, and the implementation of specific diets, such as the ketogenic diet. Of all the interventions, the more frequent interventions, 11 were dietetics supplements, while only 5 were diet interventions. The intervention more frequent was carbohydrate ingestion in four studies, followed by creatinine supplementation in three papers and tart cherry supplementation in three studies. In Table 1, we present a detailed list of studies, including author, country, design, participants, sex, intervention, control, timing, and duration of the intervention. Three studies conducted interventions during recovery following a game or training session. The remaining 12 studies were in the period prior to the measurement. In the post-studies, the mean of the treatment was one day (SD = 0.99). In the prior intervention, the average time of treatment was 14.95 days (SD = 11.11)

### 3.5. Outcome Measurements

The included studies encompassed various outcome measures, reflecting the diverse aspects of physical conditioning, muscular function, and post-exercise responses in soccer players. The authors categorized these measures into four main groups: physical conditioning tests, muscular strength, power assessments, biomarkers of exercise-induced responses, and subjective assessments. (Table 2) Researchers frequently utilized physical performance tests, including the Loughborough Intermittent Shuttle Test (LIST), countermovement jump (CMJ), sprint tests, agility runs, and Yo-Yo Intermittent Recovery tests, in their studies. These tests evaluate various aspects of soccer performance, including endurance, speed, and agility. Muscular strength and power assessments included maximal voluntary isometric contraction (MVIC) measurements, leg press, bench press, and vertical jump. These outcomes provide insights into the soccer players’ strength, power, and explosiveness.

Additionally, mean power output (MPO) and fatigue index (FI) were assessed in some studies, offering further information about the players’ anaerobic capacity and susceptibility to performance decrements, following the guidelines presented by Enoka and Duchateau [108] on the appropriate usage of fatigue terminology. Biomarkers of exercise-induced responses included oxidative stress markers, vertical ground reaction force (VGRF), electromyography (EMG) muscle activation, and energy expenditure. These markers help evaluate the soccer players’ physiological and neuromuscular responses to the interventions and their potential effects on physical conditioning and post-exercise adaptations.

Subjective assessments, such as perceived recovery and delayed-onset muscle soreness (DOMS), were also reported in the studies. These outcomes provide valuable insights into the soccer players’ subjective experiences of recovery and overall well-being. Players participated in two soccer matches in one crossover study, and the intervention group won both. This result highlights the potential impact of nutritional interventions on real-world competitive outcomes for soccer players.

Overall, the various outcome measures reported across the 16 studies emphasize the complexity of evaluating the effects of nutritional interventions on soccer performance and recovery. In Table 2, we present a detailed summary of the outcome measures employed in each study, offering an overview of the performance and recovery variables assessed.

In light of the distinct physiological demands and nutritional requirements between professional and semi-professional athletes, we have divided the Section 3 into two segments to meticulously examine and compare the impact of nutritional interventions on these divergent groups, thereby ensuring the validity and relevance of our findings to each athlete’s specific level of training intensity and body composition.

### 3.6. Studies with Professional Soccer Players

There were 12 studies performed with professional soccer players.

#### 3.6.1. Recovery Studies

Recovery in sports and exercise science refers to the time and methods athletes employ to restore their physiological and psychological functions to pre-exercise levels. Recovery is a critical period that alleviates fatigue, replenishes energy reserves, and repairs muscle damage, contributing to enhanced performance in subsequent exercises or matches. Two studies [99,106] examined various aspects of recovery in soccer players, specifically focusing on different interventions and comprehensive recovery protocols. One study [106] compared two recovery strategies after an unofficial game: one that incorporated tart cherry, cold water immersion (CWI), and foam rolling, and another that involved intermittent CWI and stretching. Although both protocols appeared beneficial for recovery, no significant differences were identified in players’ physiological, neuromuscular, or perceptual outcomes. The second study. [99] evaluated the effects of meals with high and low glycemic indexes consumed during a 22 h recovery period on performance in the subsequent Loughborough Intermittent Shuttle Test. This study also compared a high-glycemic diet (glycemic index of 70) with a diet having a glycemic index of 30 but found no significant differences. Despite these investigations, neither of the studies provided definitive evidence of the effectiveness of the examined interventions during the recovery period. These findings underline the complexity of the recovery process and indicate a need for further research to identify effective recovery strategies for soccer players.

#### 3.6.2. Performance-Based Nutritional Interventions

Eleven investigations studied performance-based nutritional interventions [15,94,96,97,98,100,101,103,104,105,107].

##### Creatine

Creatine was the focus of a crossover study [97] and three clinical trials [15,98,105], with intervention durations ranging from 6 to 14 days and doses varying from 5 g four times a day to 0.03 g/kg/day of creatine monohydrate. These studies detected significant differences in repeated sprints, agility runs, muscle activation, peak power output, and mean power output [97,98,105]. Another trial examined the combination of bicarbonate and creatine [15]. Observing significant differences in sprint performance.

##### Other Interventions

Various other interventions were studied. A clinical trial on raw pistachio nut kernels [107] found that consuming 25 g/day for 21 days positively impacted the redox status of professional soccer players compared to the control group. The study detected increases in total thiol, disulfide/native thiol ratio, and disulfide/total thiol ratio. It also detected a decrease in the native thiol/total thiol ratio. The increased total thiol suggests pistachio consumption boosts soccer players’ antioxidant capacity. The elevated disulfide/native thiol ratio indicates a shift towards a more oxidized state, enhancing the body’s ability to neutralize harmful substances. The increased disulfide/total thiol ratio implies that pistachio consumption promotes disulfide bond formation, supporting redox balance and protecting against free radicals. The observed decrease in the native thiol/total thiol ratio corroborates prior studies, indicating that pistachio intake enhances the redox status of soccer players. Increased antioxidant capacity and disulfide bond formation promotion may contribute to this improvement. Such alterations potentially affect players’ performance, recovery, health, and well-being.

Another trial [96] reported significant improvements in the Repeated Anaerobic Sprint Test (RAST) for players who ingested bicarbonate and minerals. In a double-blind clinical trial, daily ingestion of 2 g/day of betaine for four weeks positively affected layer strength as measured by bench and leg presses [100]. Conversely, a clinical trial with yohimbine for 21 days found no difference in strength compared to the placebo group [101]. A trial with a symbiotic diet found a significant increase in Kcal/week and Mets measured by accelerometry [105]. Lastly, a clinical trial comparing a high-carbohydrate diet of 8 g CHO/kg/day to a low-carbohydrate diet of 3 g CHO/kg/day [106] detected a significant increase in the distance covered, indicating the effectiveness of the high-carbohydrate diet.

### 3.7. Studies with Semiprofessionals Soccer Players

There were three studies with semiprofessional soccer players. A crossover study investigated the impact of ingesting a carbohydrate-electrolyte solution on semi-professional soccer players’ recovery and skill performance during a 90-min Loughborough Intermittent Shuttle Test (LIST) [95]. The study used both LIST [18] and the Loughborough Soccer Passing Test as outcome measures to reflect the influence of this intervention on recovery and subsequent skill performance. Despite these measures, the study did not find any improvement in soccer skill performance after the players drank the carbohydrate solution. These results are similar to another study by the same authors that did not find significant differences between the carbohydrate-electrolyte solution and the control group. [20] Tart cherry juice was used in a clinical trial [97] and showed significant differences in countermovement jump (CMJ), maximal voluntary isometric contraction (MVIC), and delayed onset muscle soreness (DOMS). A study comparing the ketogenic diet to the Mediterranean diet reported no difference in physical performance tests, such as the CMJ and the Yo-Yo Intermittent Recovery Test [104].

## 4. Limitations

The size of the studies is very small, and in some of the crossover studies, there are two studies with eight or fewer individuals [96], with a median of 18, which may lead to a lack of power and make statistically significant differences undetectable. In the future, conducting studies with more players or even multicenter studies would be interesting. A further issue is that most studies have involved male professional players. Given the increasing number of professional women’s soccer teams, conducting studies involving professional women’s soccer players is necessary. The pistachio trial had the inconvenience of not being blind because the participants knew that they ate the pistachio kernels [109].

Sixteen studies in this systematic review have utilized a broad range of measurements to comprehensively evaluate the potential impact of nutritional interventions on soccer performance. While this approach has provided a comprehensive understanding of the various aspects of soccer performance that interventions can influence, the use of various outcome measures presents both strengths and weaknesses in evaluating the effects of these interventions on performance and recovery.

In this systematic review, various outcome measures across studies offer a comprehensive insight into various aspects of soccer performance and recovery influenced by interventions. Employing a diverse range of outcome measures is a strength of intervention evaluation, as it enables a robust assessment of effectiveness, increases external validity, and accounts for individual differences, ultimately enhancing applicability to a broader range of players and situations. Specifically, various outcome measures provide a comprehensive understanding of the various aspects of soccer performance that interventions may influence.

This diversity of outcomes allows for a more robust evaluation of the effectiveness of the interventions and their potential applications in optimizing soccer performance and recovery. Including multiple outcome measures allows for a more accurate representation of the multifaceted nature of soccer performance, as different outcome measures may be more relevant to specific aspects of the sport. This diversity also helps enhance the external validity, i.e., the generalizability of the study findings to a broader range of soccer players and situations. Lastly, various outcome measures can help account for individual differences among soccer players. It acknowledges that different players may respond differently to nutritional interventions, and the variety of measures helps identify which aspects of performance are most affected by the interventions for different individuals.

On the other hand, the heterogeneity of outcome measures complicates the comparison and synthesis of results, potentially hindering definitive conclusions on the effectiveness of nutritional interventions. This diversity may introduce bias, the risk of selective reporting, and challenges in conducting meta-analyses. Inconsistency in assessment methods can affect the reliability and validity of the results, complicating the derivation of accurate conclusions. The heterogeneity of outcome measures across the studies can make comparing and synthesizing the results challenging. This variability may hinder the ability to draw definitive conclusions regarding the overall effectiveness of nutritional interventions on soccer performance and recovery. The diversity of outcome measures may introduce a risk of bias, as researchers may be more likely to report outcomes that show favorable results for their interventions. This selective reporting can potentially lead to an overestimation of the effectiveness of nutritional interventions. The wide variety of outcome measures can make it challenging to conduct a meta-analysis, as pooling data from different measures may not be feasible or appropriate. As a result, it may be challenging to synthesize the findings quantitatively and derive a more precise estimate of the overall intervention effect. Different outcome measures may also introduce variability in the methods and tools used to assess these outcomes. This inconsistency can affect the reliability and validity of the results, making it difficult to draw accurate and meaningful conclusions.

One methodological issue arises from the simultaneous evaluation of multiple interventions in two studies, which makes it impossible to discern the individual effect of each intervention [17,108]. In the future, conducting multi-arm clinical trials to test various interventions or clinical trials with a factorial design would be beneficial. Alternatively, evaluating a single measure in a clinical trial may prove valuable.

While the diverse range of outcome measures employed in these 16 studies offers a comprehensive assessment of the effects of nutritional interventions on soccer performance and recovery, the heterogeneity of these measures presents challenges in comparing and synthesizing the findings. Future research could benefit from standardizing outcome measures and adopting a more consistent approach to assessing performance and recovery variables. This would facilitate the comparison of results across studies and help provide a clearer understanding of the overall effectiveness of nutritional interventions for soccer players.

Soccer is a complex sport, with success hinged on a mix of physical capabilities, technical skills, and tactical understanding. Therefore, the enhancements achieved through nutritional supplementation may not directly translate into match performance. We should carefully consider our primary focus on athletic performance, as oversimplifying the multifaceted nature of the sport could occur when extrapolating these findings to overall soccer performance. Experimental measurement of soccer performance is challenging due to factors like athletes’ varied compliance during research compared to actual competitions, potentially affecting the reliability of findings. Furthermore, the oversight of individual differences among athletes in their response to and need for supplementation emphasizes the necessity for implementing individualized strategies, which, in turn, underscores the call for precision nutrition in sports science. The Regman study also concluded that individual approaches are most promising [109]. While our study may not detail these complexities, it acknowledges the importance of these factors and advocates for conducting comprehensive future research to determine the optimal implementation of supplementation in soccer training and competition.

## 5. Discussion

Our findings suggest that specific nutritional interventions can positively affect soccer performance and recovery. For example, creatine supplementation improves high-intensity exercise performance, while tart cherry juice may enhance recovery after strenuous exercise. Furthermore, high-carbohydrate diets seem to increase the distance covered by soccer players. However, the ketogenic diet showed no significant effect on performance measures, and the effectiveness of yohimbine remains inconclusive.

The observed improvements in performance and recovery variables may be attributed to the physiological effects of the nutritional interventions. For instance, creatine supplementation increases the creatine concentration in muscles, enhancing high-intensity exercise performance [5,6,7]. Similarly, tart cherry juice contains phytochemicals with antioxidant and anti-inflammatory effects that may aid recovery [55,56].

### Creatine

Despite the varied results from the studies analyzed in this review, it is essential to remember that many athletes, including soccer players, commonly use creatine supplementation due to its established benefits in high-intensity and intermittent exercise scenarios [99,100,107]. Creatine has been shown to enhance muscle phosphocreatine stores, improving performance in activities that involve short, intense bursts of effort [99,100,107]. Athletes in soccer and other sports have used creatine as a supplement to increase energy and reduce fatigue. The ability of creatine to enhance blood flow and oxygen delivery to muscles is a possible explanation for these effects. Recent evidence in the field of creatine supplementation has shifted the traditional approach to creatine dosing. Current research recommends lower daily dosages for effective outcomes instead of the previous high ‘loading’ doses, which often entailed 20–25 g/day for 5–7 days. The recommended dosages of 3–5 g/day or 0.1 g/kg of body mass/day are well-tolerated and effectively increase intramuscular creatine stores, muscle accretion, and muscle performance/recovery [74]. While the ‘loading’ strategy might be beneficial for athletes seeking to maximize the ergogenic potential of creatine in a short period of time, those planning to use creatine over an extended period or to avoid potential weight gain associated with creatine ‘loading’ might find the ‘maintenance’ strategy more suitable. This shift towards lower daily dosages underlines the increasing emphasis on individualized nutrition strategies in sports performance.

Furthermore, while anecdotal reports have associated creatine supplementation with muscle cramping and dehydration, current scientific evidence does not support these claims [74]. For instance, researchers evaluated injury rates in collegiate football players using creatine and found significantly less cramping, heat illnesses, and dehydration among creatine users than among non-users [110]. Creatine supplementation may even enhance tolerance to exercise in the heat, potentially reducing the risk of heat-related illnesses. Creatine can improve specific performance markers like repeated sprints, agility runs, and power output in soccer players. While creatine is generally considered safe, athletes should consult a healthcare professional or sports dietitian before starting any new supplementation regimen. As with all supplements, creatine should complement, not replace, a balanced diet and a well-structured training program.

It is necessary to design randomized double-blind clinical trials with significant sample sizes and factorial designs that study the combination of a high carbohydrate diet and bicarbonate drinks with supplements such as creatine, betaine, and tart cherry.

## 6. Conclusions

Our research sheds light on the potential of various nutritional interventions to enhance the performance of professional soccer players. It is evident that a high-carbohydrate diet, consumed in the weeks leading up to soccer matches, may positively impact performance by increasing the distance covered by players. This suggests the role of adequate carbohydrate availability in fueling prolonged physical activity such as soccer.

The ingestion of bicarbonate and mineral solutions has also been shown to enhance performance, as evidenced by significant improvements in the Repeated Anaerobic Sprint Test (RAST). This might be attributed to the potential buffering capacity of bicarbonate, which delays the onset of muscular fatigue during high-intensity exercise.

Furthermore, our findings suggest that supplements like creatine, betaine, and tart cherry may offer performance benefits. Creatine supplementation showed notable effects on repeated sprints, agility runs, muscle activation, peak power output, and mean power output, indicating its role in energy production during short, high-intensity efforts, a characteristic component of soccer. Betaine supplementation positively affected player strength, suggesting its potential role in enhancing force production. Tart cherry consumption significantly improved countermovement jump, maximal voluntary isometric contraction, and delayed-onset muscle soreness, suggesting its potential anti-inflammatory and antioxidant properties.

However, it is essential to note that despite numerous studies investigating various recovery interventions, none have proven effective in significantly accelerating or improving recovery periods in professional soccer players. This highlights the need for further research to better understand how recovery can be optimized in this population.

## Figures and Tables

**Table 1:** Summary of Included Studies: Author, Country, Design, Participants, Sex, Intervention, Control, Timing, and Duration.

Author (Year of Publication)	Country	Design	*n*	Player Category	Sex	Intervention	Control	Timing	Duration
Ali, A. (2009) [93]	UK	crossover	17	Semiprofessional	Male	6.4% carbohydrate drink to 8 mL/kg	Placebo	Post	after every 15 min of exercise
Altarriba-Bartes, A. (2023) [106]	Mexico	clinical trial	18	Professional	Male	foam roller CWI tart cherry juice 30 mL	stretching, intermittent CWI	Post	within 30 min after the end of the game and the day after
Bangsbo, J. (1992) [94]	Denmark	crossover	7	Professional	Male	65 % carbohydrate diet	39% carbohydrate diet	Pre	2 days
Bell, P.G. (2016) [95]	UK	clinical trial	16	Semiprofessional	Male	tart cherry juice (30 mL twice per day,	Placebo	Pre	7 consecutive days (4 days pre- and on each trial day
Celik, H. (2019) [107]	Turkey	clinical trial	40	Professional	Male	pistachio (25 g/day)	No pistachio control	Pre	25 days
Chycki, J. (2018) [96]	Poland	clinical trial	26	Professional	Male	Na bicarbonate, 3000 mg K dicarbonate 3000 mg Ca phosphate, 600 mg K citrate, 1000 mg Mg citrate 1000 mg, Ca citrate 400 mg	Placebo	Pre	9 days Additional doses 90 min before the exercise test protocol and the day before the test
Cox, G. (2002) [97]	Australia	clinical trial	12	Professional	Female	Creatine (5 g QID)	Placebo	Pre	6 days
da Silva Azevedo, A.P. (2019) [98]	Brazil	crossover	8	Professional	Male	Creatine monohydrate (0.3 g/kg/day)	Placebo	Pre	7 days r
Erith, S. (2006) [99]	UK	crossover	27	Semiprofessional	Male	High Glycemic Diet (GI: 70)	Low Glycemic Diet (GI: 30)	Post	22 h
Nobari, H. (2021) [100]	Iran	clinical trial	29	Professional	Male	betaine (2 g/day)	Placebo	Pre	4 weeks
Ostojic, S.M. (2006) [101]	Serbia	clinical trial	20	Professional	Male	Yohimbine (20 mg/day)	Placebo	Pre	21 days
Paoli, A.A. (2021) [102]	Italy	clinical trial	16	Semiprofessional	Male	isoprotein ketogenic diet (1.8 g/kg/day	Western diet	Pre	30 days
Quero, C.D. (2021) [103]	Spain	clinical trial	14	Professional	Male	Symbiotic Gasteel Plus^®^ (300 mg)	Placebo	Pre	1 Month
Souglis, A.G. (2013) [104]	Greece	crossover	21	Professional	Male	high carbohydrate diet 8 g CHO/kg/day	Low carbohydrate diet 3 g CHO/kg/d	Pre	3, 5 days
Yáñez-Silva, A. (2017) [105]	Brazil	clinical trial	19	Professional	Male	Creatine Monohydrate 0.03 g/kg/day	Placebo	Pre	14 days
Kim, J. (2021) [15]	Korea	clinical trial	20	Professional	Male	creatine (20 g/day) sodium bicarbonate (0.3 g/kg/day)	Placebo	Pre	7 days

CWI Cold Water Immersion. QID = four times a day, CHO = Carbohydrate.

**Table 2:** Summary of Included Studies: Author, Outcome, Magnitude, and Significance.

Author (Year of Publication)	Main Outcome	Outcome Measurements	Effect Size Intervention vs. Control	*p*
Ali, A. (2009) [93]	soccer skill performance	LIST [16], LSPT [17]	11% Performance	*p* < 0.07
Altarriba-Bartes, A (2023) [106]	physical performance	countermovement jump, hamstring maximal voluntary contraction, perceived recovery, muscle soreness	No differences	NS
Bangsbo, J. (1992) [94]	long-term, intermittent exercise performance.	total mean running distance (km)	0.9	*p* < 0.05
Bell, P.G. (2016) [95]	recovery following prolonged repeat sprint activity	LIST MVIC, 20 m Sprint * (s) CMJ 5-0-5 Agility * (s) DOMS * (mm)	No differences 19% 0.1/0.9/0.5 6% −0.1/0.5/0.2 33/46/23	NS *p* < 0.05 NS *p* = 0.017 *p* = 0.043 *p* = 0.013
Celik, H. (2019) [107]	oxidative stress caused by a strenuous soccer training program	Total thiol (μmol/L) Disulfide (μmol/L) %Disulfide/native thiol % Disulfide/total thiol % Native thiol/total thiol	4.86 9.49 1.78 1.51 −3.01	*p* = 0.015 *p* < 0.001 *p* < 0.001 *p* < 0.001 *p* < 0.001
Chycki, J. (2018) [96]	anaerobic performance	RAST	1.06	*p* < 0.001
Cox, G. (2002) [97]	performance simulating match play.	Repeated Sprints (s) Agility Runs (s) Precision Ball-Kicking	0.05 0.2 −0.3	*p* <0.05 *p* <0.05 NS
da Silva Azevedo, A.P. (2019) [98]	biomechanical parameters of running	VGRF, EMG activation intensity during the stance phase GL (au) VM (au)	1.05 1.00	*p* < 0.05 *p* < 0.05
Erith, S. (2006) [99]	performance during prolonged high-intensity intermittent shuttle running.	LIST Number attempted Sprinting Distance sprint (m) jogging to fatigue (minutes)	4 377 2.4	NS NS NS
Nobari, H. (2021) [100]	physical performance	Leg press (kg) Bench press(kg)	2.8 4.3	*p* < 0.05 *p* < 0.05
Ostojic, S.M. (2006) [101]	physical performance	Bench and leg press, vertical jump, dribble, power test, shuttle run	No differences	NS
Paoli, A.A. (2021) [102]	muscle strength, jump performance, endurance	CMJ yo-yo intermittent recovery	No differences	NS
Quero, C.D. (2021) [103]	anxiety, stress, and sleep quality	Accelerometry Kcal/week METS MVPA (min) Steps (Total/week) Sedentary bouts (>1 min)	281.8 0.06 −34.93 8309.34 −108.33	*p* < 0.05 *p* < 0.05 NS NS NS
Souglis, A.G. (2013) [104]	distances covered during soccer match	Distance covered (m) Game result (won)	1303 2 of 2	*p* < 0.01 NS
Yáñez-Silva, A. (2017) [105]	muscle power output	Wingate Anaerobic Test PPO MPO FI Total Work	5% 4% No differences 1%	*p* < 0.05 *p* < 0.05 NS *p* < 0.05
Kim, J. (2021) [15]	soccer-specific performance	10 m sprint 30-m sprint, coordination, right/left arrowhead agility, Yo-Yo intermittent recovery.	No differences −3% No differences −6.6%; −4.3% No differences	NS *p* = 0.007 NS *p* < 0.001 NS

* 24 h, 48 h, 72 h NS = No Significance; au = Arbitrary Unit; CMJ = Counter Movement Jump; LIST = Loughborough Intermittent Shuttle Test; LSPT = Loughborough Soccer Passing Test.; MVIC = Maximal voluntary isometric contraction; DOMS = Delayed Onset Muscle Soreness (assessed using a 200 mm visual analog scale (VAS) with ‘no soreness’ at one end and ‘unbearably painful’ at the other); RAST = Repeated Anaerobic Sprint Test; VGRF = Vertical Component of Ground Reaction Force; EMG = Electromyography; GL = Gastrocnemius Lateralis; VM = Vastus Medialis; MVPA = Moderate to Vigorous Physical Activity; PPO = Peak Power Output; MPO = Mean Power Output, FI = Fatigue Index.

## Data Availability

The data that support the findings of this study are available from the corresponding authors, FGG & IAO, upon reasonable request. The protocol is available at https://doi.org/10.20944/preprints202304.0261.v1, accessed on 23 May 2023.
